# Facilitators and barriers of change toward an elder-friendly surgical environment: perspectives of clinician stakeholder groups

**DOI:** 10.1186/s12913-017-2481-z

**Published:** 2017-08-24

**Authors:** Heather M. Hanson, Lindsey Warkentin, Roxanne Wilson, Navtej Sandhu, Susan E. Slaughter, Rachel G. Khadaroo

**Affiliations:** 1 Alberta’s Seniors Health Strategic Clinical Network, Alberta, Canada; 2 Department of Community Health Sciences, Cumming School of Medicine, University of Calgary, Calgary, AB Canada; 3 Faculty of Nursing, University of Alberta, Edmonton, AB Canada; 4 Department of Critical Care Medicine, Faculty of Medicine & Dentistry, University of Alberta, Edmonton, AB Canada; 5 Department of Surgery, 2D Surgery WMC, 8440-112 St NW, Edmonton, AB T6G 2B7 Canada

**Keywords:** Older adults, Post-operative surgical care, Elder-friendly, Barriers, Facilitators, Organizational readiness

## Abstract

**Background:**

Current acute care surgical practices do not focus on the unique needs of older adults. Adverse outcomes in older patients result from a complex interrelationship between baseline vulnerability and insults experienced during hospitalization. The purpose of this study is to assess the organizational readiness and the barriers and facilitators for the implementation of elder-friendly interventions in the acute care of unplanned abdominal surgery patients.

**Methods:**

This cross-sectional mixed methods study included a convenience sample of clinician stakeholder groups. Eight focus groups were conducted with 33 surgical team members including: 10 health care aides, 6 licensed practical nurses, 6 registered nurses, 4 nurse managers and 7 surgeons, to identify barriers and facilitators to the implementation of an elder-friendly surgical unit. Audio recordings of the focus groups were transcribed verbatim and analysed using interpretive description techniques. Transcripts were coded along with explanatory memos to generate a detailed description of participant experiences. Themes were identified followed by refining the codes. Participants also completed the Organizational Readiness for Implementing Change questionnaire. Differences in organizational readiness scores across clinician stakeholder groups were assessed using Kruskal-Wallice tests. Mann-Whitney tests (Bonferroni’s corrections for multiple comparisons) were conducted to assess pair-wise relationships.

**Results:**

The focus group data were conceptualized to represent facilitators and barriers to change at two levels of care delivery. Readiness to change at the organizational level was evident in five categories that reflected the barriers and facilitators to implementing an elder-friendly surgical unit. These included education, environment, staffing, policies and other research projects. At the individual level barriers and facilitators were apparent in staff members’ acceptance of new roles and duties with other staff, family and patients. Examples of these included communication, teamwork and leadership. The mean change commitment and change efficacy scores on the Organizational Readiness for Implementing Change Questionnaire were 3.7 (0.8) and 3.5 (0.9) respectively. No statistically significant differences were detected between the stakeholder groups.

**Conclusions:**

Staff are interested in contributing to improved care for elderly surgical patients; however, opportunities were identified to enhance facilitators and reduce barriers in advance of implementing the elder-friendly surgical unit intervention.

**Electronic supplementary material:**

The online version of this article (doi:10.1186/s12913-017-2481-z) contains supplementary material, which is available to authorized users.

## Background

Increasing attention is focused on the poor outcomes of hospitalized surgical patients, 65 years of age and older [1]. Current acute care practices do not focus on the unique needs of this population, yet as the Canadian population ages [2] there will be greater demand for emergency surgical services. Such challenges can result in post-operative complications [3], higher care needs post-discharge [4–6], and reduced quality of life [7].

Adverse outcomes in the older patient result from a complex interrelationship between baseline vulnerability and insults experienced during hospitalization, including fasting to promote gastrointestinal healing, polypharmacy, immobility, nasogastric tubes, and bladder catheterization [3]. In Alberta, retrospective analyses found that more than 60% of older patients developed at least one in-hospital complication, which was also an independent predictor of mortality [5]. In recognition of the room for improvement, there have been recent calls made to address hospital environments in order to improve the health outcomes for the older adult population [8]. In light of this call and given the outcomes of elderly surgical patients, we propose an alternative to the current post-operative approach to care is required.

Elder-friendly interventions embedded in the routine practice of the hospital environment and introduced during the acute phase of older adults’ illness or injury can improve patient- and system-level outcomes [9]. Furthermore, evidence suggests that an interdisciplinary approach to surgical care can improve outcomes for people of all ages, including older adults [10–13]. Work has been initiated within a major surgical centre in Alberta to develop a dedicated, interdisciplinary, elder-friendly surgical unit. Currently under development, it is expected to realign existing resources, implement evidence-based practices, and improve health outcomes in a cost-effective manner. The interventions of this initiative are targeted at acute abdominal surgical patients, 65 years of age and older, and are conceptualized to include: 1) co-locating elderly surgical patients on one unit; 2) interdisciplinary team-based care; 3) implementation of evidence-informed practices including early mobilization, delirium detection, and comfort rounds; and 4) early discharge planning to support safe, quality discharge transitions to maximize functional recovery [14].

Barriers to the uptake of best evidence are diverse, leading to unpredictable transfer of clinical and health service research findings into practice [15]. Wallace and colleagues [16] found knowledge, behaviours, and attitudinal beliefs to be key barriers to uptake of research findings. Further, contextual factors, such as environmental pressures for improvement, organizational leadership, and local culture, have been reported to influence success in quality improvement initiatives [17].

Organizational readiness for change may influence new initiatives introduced within the acute care setting. The theory of organizational readiness for change conceptualizes organizational readiness to include two facets or domains: change commitment and change efficacy [18]. Change commitment is a shared resolve among organizational members to implement a given change; while change efficacy is the members’ shared belief in their capacity to make the changes [18]. Change efficacy involves organizational members’ explicit knowledge of what to do and how to do it, perception that resources are available to implement the change, and perception that situational factors, such as timing, are favourable [18]. Together, change commitment and change efficacy comprise organizational readiness [18].

To maximize the opportunity for success of the future intervention, we elected to follow Lanham’s [19] suggestion of soliciting input into the intervention design and implementation from clinician stakeholder groups impacted by the intervention. Before the implementation of an elder-friendly surgical environment in a major hospital centre, we opted to understand staff members’ current perceptions of barriers and facilitators to the implementation and organizational readiness for change. If staff are part of the proposed future intervention, they should be included in how the intervention is to proceed [19]. An understanding of the clinician stakeholders’ views of the elements of their local environment that might influence the effectiveness of the intervention [15] was sought to ensure that the design of the intervention was based on an assessment of the factors that would support or hinder the intended health and health system outcomes [20].

As such, this article reports the perspectives of clinician stakeholders on the potential facilitators and barriers, and organizational readiness for implementing an elder-friendly surgical unit. The objective of our study is to inform the design and implementation of a larger initiative on elder-friendly interventions to improve post-operative healthcare practices and health outcomes.

## Methods

### Setting and participants

We employed qualitative focus groups with clinicians to explore their perspectives on the barriers and facilitators to change that would influence their readiness to implement change. We recruited a convenience sample of clinician stakeholders to participate in focus group discussions between June 2014 and August 2014. The stakeholder groups were conceptualized as those individuals who can affect, or are affected by, the larger project [21], and included general surgeons, registered nurses (RNs), licensed practical nurses (LPNs), health care aides (HCAs), and nursing care managers. Surgeons were invited to participate if they were a part of the Acute Care and Emergency Surgery service. All other participants were recruited from three general surgery nursing care units located at the University of Alberta Hospital, a single tertiary care centre in the province of Alberta, Canada. The study received approval from the Health Research Ethics Board at the University of Alberta and all participants provided written informed consent.

### Focus group organization

The focus groups were organized according to professional designation in order to minimize bias related to power imbalance within mixed groups. The exception to this rule was the nursing managers’ focus group, which included the three unit managers and a nursing supervisor, who would be considered their superior. On the morning of the scheduled focus group, potential participants were approached, the study was explained, and consent was obtained. Where opportunity allowed, the focus group was scheduled to coincide with a pre-existing meeting. The average duration of the focus groups was 30 min.

Data were collected through a combination of audio recordings, flip chart notes and questionnaires. The focus group discussions began with the facilitator briefing participants on the four proposed elder-friendly interventions, which were summarized on a poster. Then, using a structured focus group guide (Additional file 1), the facilitator led the group in a discussion soliciting the participants’ ideas regarding the facilitators and barriers to incorporating the proposed interventions on the nursing care units where they worked. Assessing both the barriers and facilitators was conceptualized as important in identifying the pivotal areas for attention (barriers) as well as the positive actions and environmental attributes to reinforce (facilitators). The focus group guide was designed for this purpose. Audio recordings were transcribed verbatim, checked for accuracy, using the flip chart notes as a reference, and subsequently de-identified.

### Questionnaires

At the end of each focus group, participants completed a demographic questionnaire and the Organizational Readiness for Implementing Change questionnaire [22]. This latter questionnaire, based on Weiner’s theory of organizational readiness [18], measures the domains of change commitment (5 questions averaged) and change efficacy (7 questions averaged) on a 5-point Likert scale [22]. A low score for change commitment suggests that organizational members have some resistance to change; while a low score for change efficacy suggests that organizational members perceive that the environment is not supportive of change. This Organizational Readiness for Implementing Change questionnaire has been psychometrically assessed by its developers. It demonstrated good item fit for change commitment and change efficacy using factor analysis, high inter-item consistency, and good inter-rater reliability and agreement [22].

### Analysis

Interpretive description was used to guide the analysis of the focus group transcripts. Interpretive description is a qualitative approach found in nursing scholarship to explain clinical phenomena [23]. During the initial phase of data analysis, transcripts were read to immerse the researcher in the data and to gain a holistic sense of the material. After this step, coding began and, along with explanatory memos, a detailed description of experiences of the participants was developed [23]. Broad themes were identified, which was followed by refining of the codes, and modifying where necessary, to best represent the data. NVivo 10 software (QSR International, 2012) was used to manage data during the analysis.

Questionnaire responses were calculated as mean (standard deviation) for continuous data and count (proportion) for categorical data. Kruskal-Wallice Tests were used to assess the difference in scores across clinician stakeholder groups. If the *p*-value of the Kruskal-Wallice test was <0.05, Mann-Whitney tests (corrected with Bonferroni’s method for multiple comparisons) were conducted to assess pair-wise relationships. STATA software version 12 (StataCorp., 2011) was used for the statistical analysis.

## Results

### Demographic characteristics

Within the three hospital wards where recruitment took place, 33 of the 36 staff members approached agreed to participate (92% response rate), including 10 HCAs, six LPNs, six RNs, seven Surgeons, and four Managers (three Unit Managers and one Patient Care Manager or supervisor). Focus groups ranged in size from four to six participants. A total of eight focus groups were conducted, with two focus groups for each of the HCA, LPN, and RN stakeholder groups. Participant demographics are summarized in Table 1. In brief, the majority of participants were female (82%), worked full-time on the day shift for a mean (SD) of 10.4 (8.6) years.

**Table 1:** Demographic characteristics of study participants

Stakeholder Group	N	Response rate^a^ (%)	Female N (%)	Years in role Mean (SD)	Years with this surgical service Mean (SD)
Health Care Aides	10	100	10 (100)	10.9 (10.0)	6.5 (5.2)
Licensed Practical Nurses	6	86	5 (83)	9.9 (8.1)	6.9 (6.7)
Registered Nurses	6	75	6 (100)	6.8 (8.3)	5.7 (6.6)
Surgeons	7	100	2 (29)	15.1 (8.2)	8.7 (3.8)
Nurse managers	4	100	4 (100)	7.4 (5.5)	13.7 (14.6)
Total	33	92	27 (82)	10.4 (8.6)	7.8 (7.1)

^a^response rate = agreed / approached

### Facilitators and barriers to change: Two levels of care delivery

**Fig. 1:**
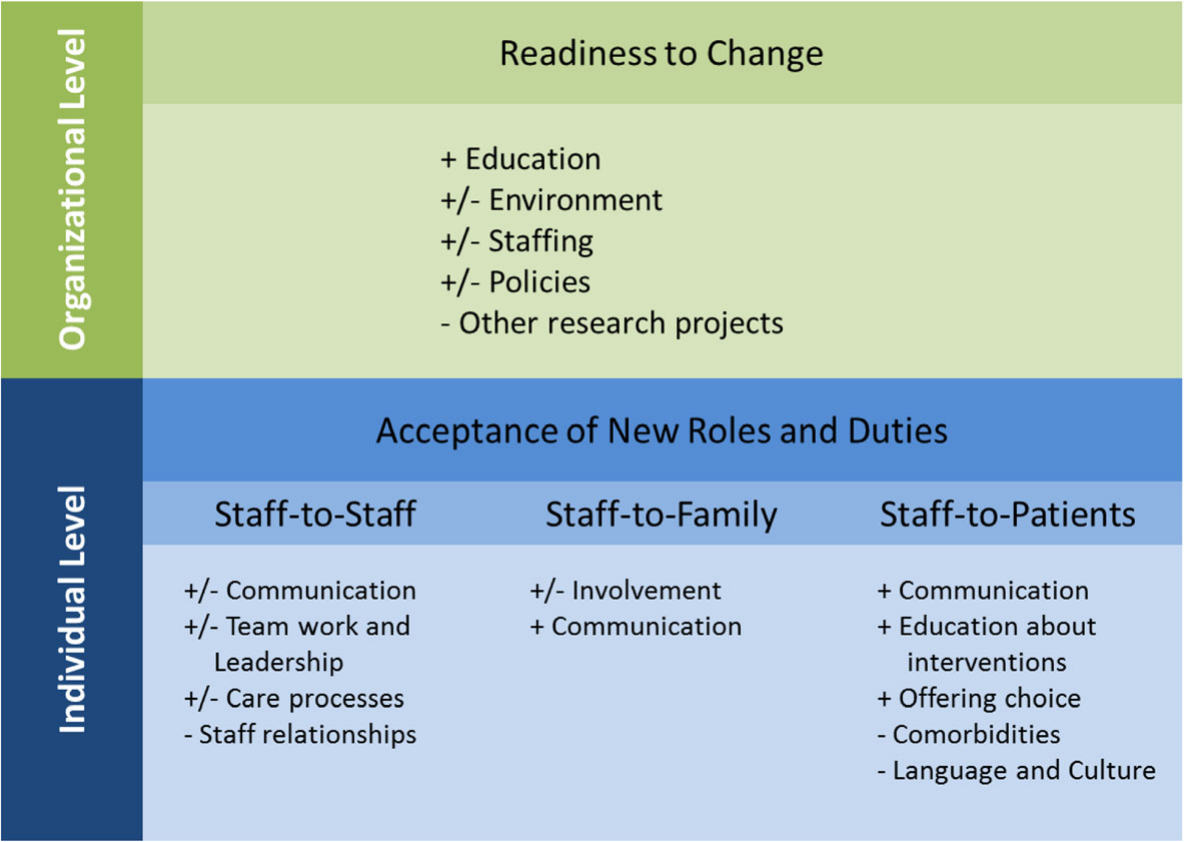
Facilitators and barriers to change by levels of care delivery. Legend: Symbols indicate (+) Facilitator; (−) Barrier; (+/−) Facilitator and Barrier

Focus group participants described barriers and facilitators for implementation of an elder-friendly surgical environment. Data were characterized at two levels: the organizational level as “readiness to change” and the individual level “acceptance of new roles and duties”. The themes and subthemes identified at the organizational and individual level are discussed separately below. These findings are depicted in Fig. 1 and summarized in Table 2.

**Table 2:** Summary of Stakeholder Comments

Stakeholder Participants	Comments: Barriers & Facilitators
Organizational Readiness for Change	
HCAs	• Making environment elder friendly with mirrors & intercoms • Environmental barriers: unsuitable unit layout & insufficient space
LPNs	• Environmental barriers: slippery floors, low toilet seats & absence of railings
RNs	• Staff shortages
Nurse Managers	• Policy on admission assessment - basic information needs
Surgeons	• Staff shortages • Staff and Physician education • Too many overlapping research projects
Individual Acceptance of New Roles and Duties	
HCAs	Staff-to-staff level • Inappropriate care culture like passing on care work from one health worker to another • Negative staff relationships: HCAs perceive heavy tasks assigned to them by RNs and LPNs Staff-to-family-level • Involvement of family in patient care • Improving communication with family using story boards Staff-to-patient level • Offering choice in care: e.g. bathing, clothing, meals • Patient language and culture barriers
LPNs	Staff-to-staff level • Effective communication strategies: problem boards • Improving teamwork Staff-to-family-level • Involvement of family in patient care Staff-to-patient level • Comorbidities: lead to difficulty predicting discharge date • Comorbidities: lead to more work
RNs	Staff-to-staff level • Effective communication strategies: communication book, report, whiteboard, clipboard etc. • Insufficient communication due to casual staff • Improving teamwork • Negative staff relationships: conflict with HCAs regarding delegation of work Staff-to-family-level • Lack of family involvement in patient care Staff-to-patient level • Improving communication strategies with patients about their progress • Educating patients about interventions • Comorbidities: involve more work and time requirements
Nurse Managers	Staff-to-patient level • Improving communication strategies with patients about their progress and treatment options
Surgeons	Staff-to-staff level • Improving teamwork • Insufficient communication among interdisciplinary professionals • Inappropriate care culture: unfocused geriatric consultations Staff-to-family-level • Improving communication with family through consistently scheduled health care professional rounds

#### Organizational level: Readiness to change

Within the major theme, organizational readiness to change, five categories emerged that reflected the barriers and facilitators to implementing an elder-friendly surgical unit at the organizational level. Education, environment, staffing, policies and other research projects were identified as barriers and facilitators to the implementation of an elder-friendly surgical unit.

##### Education

The organization plays a key role in arranging for and imparting education about new initiatives. Education on the changes included as part of the intervention was considered important in facilitating implementation by the participating stakeholder groups. Participants felt that not everyone is being well educated when something new is started within the hospital, with one surgeon commenting on the need for “educating the frontline staff as well as physicians”.

##### Environment

Participants reported that improvements to the environment are under the control of the organization. The HCA group, in particular, talked about the environment as a factor that acts as a facilitator and barrier to the implementation of elder-friendly interventions. As a facilitator, they suggested use of mirrors or intercoms for improved coverage of the units, to the benefit of visibility and awareness of what is happening elsewhere on the unit. As a barrier, they specifically pointed to the lack of sufficient space and were dissatisfied with having 3 beds in a 2 bed designed room. They were also not happy with the layout of the unit and cited the example of a HCA attending one patient and not hearing a fall experienced by a patient on the other side of the unit. Further, the LPN groups felt that the floors, “are too slippery for the elderly, it’s hard to walk.” They were also concerned about the low toilet seats and lack of railings in bathrooms to grab for support. They suggested raised toilet seats, more commodes with handles, and railings on both sides of the bathrooms to address these barriers.

##### Staffing

All groups considered staffing issues as a significant organization-wide barrier to implementing an elder-friendly surgical unit. Surgeons perceived that there was insufficient weekend ancillary staff coverage, including a geriatric consultation service, and were sceptical of seeing the intervention changes incorporated by casual staff. The RN group reported that staff shortages already lead to feeling overwhelmed and suspected that staff would forget or leave out certain aspects of the interventions when busy, considering them as “not really important.” They also mentioned that with short nursing staff and insufficient interdisciplinary staff, co-locating elderly patients to one unit would be burdensome and described such a unit as “heavy.”

##### Policies

Policies at the hospital were cited as a barrier to implementing the elder-friendly surgical unit. The manager group identified the need for establishing a minimum information requirement for taking patient histories. Complete information needs to be collected for efficient care delivery and discharge planning, but the unit was reportedly lacking complete information since the move to an electronic health record. The manager group felt that obtaining baseline information from patients, such as their mobility status, disabilities, home situation, and community services they were already receiving, would be a significant improvement, given that, “the nurses are supposed to review it with the patients and make sure that the information is complete and accurate.”

##### Other research projects

Some groups identified the presence of other research projects as a barrier to the implementation of new initiatives. They anticipated feeling overwhelmed and confused with the different guidelines and protocols of the various projects taking place at the hospital. A surgeon described, “There’s lots of resistance. There are many overlapping protocols and processes going on right now.”

#### Individual level: Acceptance of new roles and duties

Participants identified facilitators and barriers that can be conceptualized at the individual level. These facilitators and barriers are dependent on staff willingness to accept intervention-related roles and care tasks, where a lack of acceptance of new roles and duties would hinder the intervention implementation while acceptance would facilitate implementation. The barriers and facilitators have been identified at the staff-to-staff level, staff-to-family level, and staff-to-patient level (Fig. 1).

### Staff-to-staff level

#### Communication

All groups identified effective communication as a facilitator to elder-friendly interventions. The RN group suggested different strategies for promoting communication among staff, such as using a communication book, report, whiteboard, or clipboard. The LPN group advised having a problem board to improve communication, “so any concerns that we have we put it on the problem board, and then…so when they come in, they take a look at the problem board and then address those.”

Insufficient communication was identified by almost all groups as the main barrier to implementing an elder-friendly surgical unit. The surgeon group reported a lack of sufficient communication and collaboration among different interdisciplinary professionals. They found it difficult to get back to the main person dealing with a specific patient, and called the current system of interdisciplinary work as, “siloed”. The RN group reported challenges when staff members do not work regularly, citing, “we are not all full-timers and we don’t work every day…sometimes you come back and things change and nobody says anything, and you’re left in limbo.”

#### Teamwork and leadership

The surgeon, RN and LPN groups reported that improved teamwork would help to sustain the elder-friendly interventions. This included better communication and improved shared care between the HCA and RN groups. One RN gave an example of the shared responsibilities with the RNs being more involved in walking and assisting patients to eat.

To promote teamwork, one HCA suggested having an HCA as a team leader who is dedicated only to seniors, “Have like a team leader come around and see how we’re doing.” Many of the stakeholder other groups also emphasized the importance of a strong leader in implementing the interventions. In order to avoid conflict between nursing groups, suggestions were made to assist with clear delegation of tasks and championing the intervention.

#### Care processes

Participants also considered the approach to care to be a barrier to the implementation of the proposed interventions. For example, surgeons thought the geriatric consultations were unfocused and did not include timely follow-up. They stated that the geriatricians “order a bunch of blood work then they disappear for another week.” Similar dissatisfaction with the current approach to care and the current culture of care delivery was identified by the HCA group. They reported that a lot of “passing on” happens, especially in the case of elder care, where tasks are passed from one health worker to another and later left forgotten.

#### Staff relationships

The relationships between staff and the perceptions of staff towards each other were identified as staff level barriers. The RN group recalled conflict when delegating work to HCAs. One RN commented that the HCAs should show initiative and not rely on the RN always having to direct them to complete tasks. The HCA group felt that the hard physical work of the elder-friendly intervention would fall to them, with the RNs and LPNs sitting back doing nothing or passing the heavy tasks to them. The HCA group reported a significant desire to change attitudes because of the existing power relations they felt were present between provider groups. Exemplifying these dynamics, one HCA stated, “Even if we do say something it still doesn’t make any big difference, ‘cause by the end of the day it’s our words against the RNs.”

### Staff-to-family level

Many participants emphasized the important role that family members play in the care of elderly patients. Various facilitators and barriers at this level were identified by the participants.

#### Involvement

Family involvement can assist the care staff by providing sufficient background information on lifestyle, nutrition, and abilities to aid in discharge planning. Family involvement is particularly helpful when problems such as delirium and pain co-occur, and the family is able to provide a pre-hospitalization reference point. The LPN group commented that at times the family members help in mobilizing and assisting patients to eat, especially if they visit around mealtimes when the unit is busy. The HCA group suggested that family involvement helps to ease the patients’ feelings of being institutionalized by bringing in personal belongings such as the patient’s own clothes and other items to make the environment feel more home-like.

Lack of family involvement can be a barrier to transition optimization. The RN group reported this barrier in relation to discharge planning, where, “if there’s not family support it makes it a little harder to get them home.”

#### Communication

Participants suggested some ways to improve communication with families. The surgeon group suggested having timed rounds of the physicians and other interdisciplinary team members to enhance interaction with family:

“They will know when the doctor will be around and the team will be around so timing will bring the family into the interdisciplinary team base care as well as into the discharge planning.”

The HCA group recommended communicating with families through the use of story boards, which would provide information on likes and dislikes that the care staff might find useful, referencing a previous positive experience, “we had a long term patient in room 6…And we did a story board for her…She liked to be called Anne instead of Annie.” They also reported communicating with families regarding mobilization expectations.

### Staff-to-patient level

As the central focus of care delivery, patients were thought to play a role in the implementation of elder-friendly interventions. Patients’ willingness to accept or reject the intervention strategies would influence implementation of these interventions.

#### Communication

The RN group cited that involving cognitively able patients in their own care would act as a facilitator. They suggested involving patients in communicating their own progress through recording activities on charts or sheets, such as recording fluid intake. Consistent with involving family as part of the interdisciplinary care team, it was also suggested that cognitively able patients also participate as an active member of their own care team. The manager group suggested clearly communicating postoperative mobility expectations to patients and encouraging patients to integrate these practices, such as walking, into their routines.

#### Education about interventions

The RN group suggested educating patients on elder-friendly practices. If there was appropriate education to the patients on the expectations then the RN group felt it would be easier for them to implement. One RN responded in support of educating patients:

“It’s easier to mobilize and it’s easier to tell [the patient] they can go to the bathroom on their own…when [nurse educators] do the teaching part when you’re coming in it still is in [the patient’s] mind.”

#### Offering choice

Providing patients with choice was cited as a facilitator to providing elder-friendly care. For example, the HCA group suggested providing meal choices to patients could improve nutrition and encourage independent eating. Soft and easy-to-swallow foods could be provided as an option to daily pureed foods. They also felt that offering patients choice in many other areas (clothing, bathing, and other care decisions) could improve overall post-surgical well-being.

#### Comorbidities

Patients’ comorbidities were cited by many stakeholder groups as being a barrier to an elder-friendly surgical unit. The RN group commented that the newly designed unit would have a patient population with a high degree of frailty and dementia, and, as such, would require more time, “some people are extremely hard to mobilize or they won’t eat because they’re dementia patients, and you need to spend more time with them.” The LPN group mentioned that due to the complex history and disease states, it is hard to know about the exact day of discharge, which might present challenges for discharge planning and transition optimization. The LPN group expressed that this unit would require more lifts, turning, continence product changing, and drug administration, all of which take time.

#### Language and culture

Finally, the HCA group reported that some other patient characteristics might be barriers to the implementation of interventions. They considered the impact of language barriers and different cultural norms to be patient-specific challenges to the implementation of interventions.

### Organizational readiness for implementing change questionnaire

**Fig. 2:**
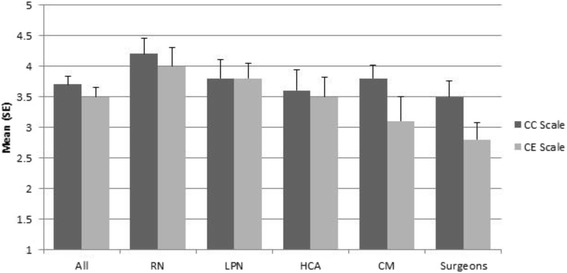
Organizational Readiness to Implementing Change questionnaire responses, as provided by clinician stakeholder groups^a^. Legend: ^a^ *N* **=** 33; Registered Nurses (RN), *n* = 6; Licensed Practical Nurses (LPN), *n* = 6; Health Care Aide (HCA), *n* = 10; Care Managers (CM), *n* = 4; Surgeons, *n* = 7. CC Scale = Change Commitment Scale; CE Scale = Change Efficacy Scale. Higher scores indicate greater agreement

The mean change commitment (CC Scale) score was 3.7 (0.8) and the mean change efficacy (CE Scale) score was 3.5 (0.9) (Table 2). The staff agreed most with the change commitment question regarding “wanting to implement change” (4.0 [0.92]) and they agreed least with the question regarding “doing whatever it takes” (3.4 [1.0]). Change efficacy was rated lower overall, compared to change commitment. The staff agreed most with the change efficacy question regarding “getting people invested,” “being supported through the change,” and “handling challenges” (3.6 [1.1], 3.6 [1.2], and 3.6 [1.1], respectively). The staff agreed least with the question regarding “managing the politics” (3.1 [1.2]). There were no statistically significant differences between the stakeholder group scores (Fig. 2).

## Discussion

The comments from the focus group participants were represented by two conceptual themes: readiness to change, which encompassed organizational-level factors that were thought to impact implementation of the proposed elder-friendly interventions, and acceptance of new roles and duties, which encompassed individual-level factors and individuals’ interactions with other staff, families, and patients. To our knowledge this study is the first to report using the Organizational Readiness to Implementing Change questionnaire [22]. Collectively participants reported highest scores on the Organizational Readiness to Implementing Change questionnaire for the change commitment item “wanting to implement change”, and lowest scores for the change efficacy item “managing the politics”.

We observed some convergence between the focus group and the questionnaire data sources. The high score for “wanting to implement change” coincided with the focus group data in which participants expressed a strong desire to see improvements in the care of elderly surgical patients. Likewise the low score for “managing the politics” coincided with focus group data demonstrating participants’ concern about the hierarchy entrenched across the HCA, LPN, and RN staff relationships and overlapping protocols across various new hospital initiatives. The focus group data provided a deeper understanding of the questionnaire findings.

### Implications for future planning

Given that the purpose of this work was to assess organizational readiness for change and the associated barriers and facilitators for implementation of the elder-friendly surgical environment, we discuss the implications of the findings for planning the implementation in relation to the four components of the intervention: co-location of elderly acute abdominal surgical patients, interdisciplinary team-based care, implementation of evidence-informed care practices, and early discharge planning and transition optimization.

The first component of the proposed intervention is the co-location of elderly acute abdominal surgical patients. Focus group participants raised concerns about increased physical care demands of older adults with a high degree of frailty and cognitive impairment. Co-locating older patients within the proposed intervention will necessitate a focus on patient centred care which includes understanding how to support the surgical recovery of patients with post-operative delirium, dementia or both. In-service training, such as a geriatric care refresher course, might be one solution to increase knowledge and awareness of frequently occurring problems for hospitalized older adults.

The second component of the proposed intervention is interdisciplinary team-based care. One of the issues related to this intervention was the frequency in which specialist skills are available on the unit. Participants referenced geriatricians, pharmacists, occupational therapists, and social workers as among the health professional groups where the availability or frequency of consultation was felt to be insufficient, to the disadvantage of an integrated, interdisciplinary team-based approach. The fact that stakeholders have identified the issue and raised it during the focus group as an area requiring attention, suggests receptivity to interdisciplinary team-based care. A recent narrative review described and summarized outcomes of innovative models of care for the management of older adults with hip fracture [12]. Specifically, co-leadership (geriatrician and surgeon) or a geriatrician led fracture service showed a tendency toward better patient outcomes including in-hospital complications, functional status recovery and in-hospital mortality. This literature and our findings support the importance of including a geriatrician along with the geriatric consultation service as part of the postsurgical care team.

However, despite receptivity to interdisciplinary team-based care, a major challenge to this approach is the existing power dynamics within stakeholder groups. Others have reported workforce issues and lack of inter-specialty collaboration as barriers to establishing perioperative geriatric medicine services [24]. The power imbalance between health professionals has been identified as a barrier to interdisciplinary collaboration [25], and was both implicitly and explicitly evident in the focus groups. Some stakeholders reported significant passing of tasks down the nursing hierarchy, and the perception of some groups that other groups are content to sit back and do nothing. Perhaps related, there were also concerns expressed regarding confusion about job expectations, roles, and delegation of tasks. One strategy moving forward may be to emphasize a unit culture in which elder-friendly care is everyone’s responsibility. In relation to scale up and spread of effective interventions, Lanham et al. [19] reported that the degree of interrelatedness among project team members can impact the successful uptake of an intervention, and in the case of interdisciplinary care in a clinical setting we argue the same may hold true. All staff, not just one provider group, must feel responsible for and committed to the success of the interventions and see themselves as an integral team member in achieving that success. HCAs and LPNs must be viewed as part of the interdisciplinary team, with their perspectives and information valued. Being listened to by other clinician stakeholder groups would go a long way to building more effective relationships within the team. Further, given the fact that HCAs have extensive day-to-day interactions with patients, they are in a strategic position to build relationships between patients, their family members, and the care staff on the unit.

Participants suggested the inclusion of patients and families as members of the interdisciplinary team. This approach aligns well with the values underpinning patient-centred care, [26] and could have many advantages. Family members of this population are likely already caregivers in some capacity, and formalizing their role as a team member would demonstrate an appreciation of their experience with, and knowledge of, the patient’s care needs. Families can be a source of background information when taking histories, which may aid in situations such as delirium detection. They can also contribute with care provision, offering an extra set of hands during high-demand times such as change of shift, and provide valuable insight and information when assisting in care plan development. In addition to including family as a part of the care team, increasingly, patients themselves are being included. Shared decision-making among the patient and their healthcare team has been successful in addressing the barrier of insufficient time by including able patients as part of the team during interdisciplinary rounds [27]. Nadler and colleagues [28] found that multi-disciplinary team meetings were essential for both initial adoption and adherence to a post-operative guideline. Finding practical methods and opportunities for team building and enhancing team communication would be an important intervention to optimize interdisciplinary team work.

The third component of the proposed intervention is implementation of evidence-informed care practices. Three key evidence-informed care practices planned for the implementation include early mobilization, delirium prevention and detection, and comfort rounds. However, given that participants reported already feeling stretched, care must be taken when introducing and implementing these care practices within the elder-friendly surgical unit. Participants reported that patients’ acute issues and medical instability come first, so when the unit is hectic or short staffed, these proactive care practices can be left undone or forgotten. Education is needed to increase understanding of the importance of simple interventions, such as mobilization, in post-surgical recovery so that such interventions can be integrated into routine practice and emphasised as an essential part of, and not superfluous to, good surgical care. Participants also raised concerns regarding the policies and procedures which result in some tasks being repeated while others missed. This is particularly relevant to other care transformation and research initiatives taking place within the hospital. High fidelity of the elder-friendly surgical unit interventions will necessitate reconciliation of the discrepancies or inconsistencies of tasks and practices common across initiatives. Any additional burden on staff must also be considered prior to implementation.

The final component of the proposed intervention is early discharge planning and transition optimization. Participants cited the complex medical histories and comorbidities of patients as a barrier to early anticipation of a discharge date and location. Missing information when patients arrive on the unit was identified as a challenge, particularly related to the potential suitability of discharging the patient home. Patient and family dynamics were raised by participants in this study. Families’ expectations around the patient’s suitability to return home following surgery were cited as a challenge. Nadler and colleagues [28] also found that the expectations of patients and their family members were barriers to early discharge planning. Nadler et al. reported patient factors as a major determinant of patient ambulation post-operatively which had an influence on length of stay. Although not expressed by the surgeon stakeholder group in this work, the perceptions of Nadler et al.’s surgical residents parallels the other stakeholder groups here, suggesting that the staff-to-patient sub-theme uncovered in this study may play a larger role in early discharge planning than would be expected in other clinical populations.

There are several strengths of this study. Most (92%) of the stakeholders approached agreed to participate in the study. This suggests the representativeness of ours the findings to the other clinicians working on the participating surgical units. The safe environment created during the clinician stakeholder focus groups appears to have enabled participants to speak their minds. For examples HCAs expressed less-than-desirable comments regarding delegation of the ‘scut work’ while others sit behind the nursing desk. Such open and honest dialogue during the focus groups should translate to an accurate assessment of the major barriers and facilitators, thus improving the opportunity for success of the intervention once these findings are integrated into the planning and implementation of the intervention. Our findings may have transferability to other acute care settings considering interventions, where readiness for change and acceptance of new roles and duties would be important factors for consideration in the design and implementation of practice changes.

A limitation of the study was the absence of key stakeholder groups other than nursing staff and surgeons. Other stakeholder groups could have included administrators, primary care physicians and interdisciplinary consultants such as physical therapists, occupational therapists and pharmacists; especially when the proposed change is to expand the interdisciplinary approach of a surgical unit. Geriatricians are another key interdisciplinary stakeholder group for an elder-friendly surgical unit. One review of the care and management of orthopaedic surgery units demonstrated better outcomes with geriatrician leadership or co-leadership (geriatrician and orthopaedic surgeon) compared with traditional leadership models [12]. Other researchers have emphasized the critical importance of soliciting patients’ perspectives of the facilitators and barriers to implementing an enhanced approach to post-surgical care [10].

We assessed stakeholder groups’ perceptions of barriers and facilitators using a questionnaire without directly observing the barriers and facilitators. However, Thomas and Thomas [29] argue that if individuals define their situation as real, the situation is real in its consequences. This supports a focus on staff perceptions, particularly when we used both qualitative and quantitative methods to elicit the perceptions. Further, Shea and colleagues [30] highlighted the importance of attending to the perceptions of clinicians and providers, as their perceptions can impact subsequent implementation activities. This work gives voice to the concerns and suggestions of those directly impacted by the new intervention, fostering a respectful relationship between the researchers and clinicians involved.

We agree with Bostrom and colleagues’ [31] suggestion that knowledge translation interventions in the care of older adults are fertile ground for further research. We plan to carefully implement knowledge translation and behaviour change strategies to address the areas for attention identified in this work. While this study specifically addressed barriers and facilitators to elder-friendly post-surgical care, elder-friendly principles are not limited to this care context. We echo the call made by Parke and colleagues [8] for a commitment to an elder-friendly hospital vision, with the challenge to extend elder-friendly approaches across the entire hospital organization.

## Conclusions

This study aimed to gain insights from clinician stakeholders on the barriers, facilitators and organizational readiness for implementing an elder-friendly surgical unit. The findings highlight two major points. First, staff are engaged and interested in contributing to improved care for elderly surgical patients. Clinician stakeholders’ participation in the present study is early evidence of receptivity to, and readiness for, organizational change to achieve the proposed interventions. However, there are areas that necessitate attention in advance of implementing the elder-friendly surgical unit, which is the second major point emerging from this work. Targeted areas for attention should include fostering an effective interdisciplinary team approach, enhancing communication regarding both the intervention-related tasks and the associated job roles and expectations, and offering education on the importance of the intervention-related care practices. While education is good, it does not, in itself, lead to behaviour change [32]. Therefore, many different approaches will be necessary to see unit-wide behaviour change related to the intervention. Given that readiness to change is a dynamic construct and can change over time [33, 34], our goal is to use the information obtained from our study to improve the environment, thus supporting successful implementation of the new elder-friendly surgical unit. Consideration will be given to reassessing readiness following the implementation of the intervention.

## Additional file

Additional file 1: “EASE STUDY Semi Structured Focus Group Guide.” This guide was used to navigate discussion in each focus group. (DOC 35 kb)
