# Supplementary material for: Facilitators and barriers of change toward an elder-friendly surgical environment: perspectives of clinician stakeholder groups
Source: BMC Health Serv Res. 2017 Aug 24;17:596. doi: 10.1186/s12913-017-2481-z (PMC5571616; doi:10.1186/s12913-017-2481-z)
Supplement: Additional file 1: — “EASE STUDY Semi Structured Focus Group Guide.” This guide was used to navigate discussion in each focus group. (DOC 35 kb) [file 12913_2017_2481_MOESM1_ESM.doc]

**Additional file 1**

**Semi-Structured Focus Group Guide**

**A. Obtain Informed Written Consent**

**B. Complete Demographic Information Sheet and Questionnaires**

**C. Focus Group**

**Assessment of PRIHS Grant Elder Friendly Interventions**

1. The Elder Friendly interventions proposed in the PRIHS grant include:
   1. Collocating high risk elderly surgical patients on the same nursing unit
   2. Interdisciplinary team based care including: geriatric consultants, nurses, rehabilitation providers, pharmacists, dieticians and social workers
   3. Evidence informed practices: medication review & reconciliation; early mobilization; prevention of post-operative complications; avoidance or early elimination of tubes; *Comfort Rounds* including: support of mobility, optimal nutrition and hydration, pain management, delirium prevention/management; and education of patients/families/healthcare providers
   4. Care transition optimization to support safe quality discharge. This coordinated plan will be established at admission and will include appropriate discussions with the patients and caregivers.

**Which of these interventions would you say could make a surgical unit Elder Friendly?**

**Prompt: Why?**

1. **If you had to choose only one of these changes to make the unit more Elder Friendly which would you choose?**

**Prompt: Why?**

**Barriers and Facilitators to Elder Friendly Interventions**

1. **What would make it difficult to implement the suggested Elder Friendly interventions?**

**Prompt: Why?**

1. **What would make it easier to implement the suggested Elder Friendly interventions?**

**Prompt: Why?**

**Suggestions for Elder Friendly Interventions**

1. **What other interventions could you suggest to make a surgical unit an ‘Elder Friendly’ surgical unit?**

**Prompt: What else could the team do to make the unit more responsive to the needs of older adults?**
